# FiO_2_ Before Surfactant, but Not Time to Surfactant, Affects Outcomes in Infants With Respiratory Distress Syndrome

**DOI:** 10.3389/fped.2021.734696

**Published:** 2021-10-04

**Authors:** Piotr Kruczek, Paweł Krajewski, Roman Hożejowski, Tomasz Szczapa

**Affiliations:** ^1^Department of Neonatology, Ujastek Medical Center, Cracow, Poland; ^2^Department of Neonatology, University Center for Mother and Newborn's Health, Warsaw, Poland; ^3^Medical Department, Chiesi Poland, Warsaw, Poland; ^4^Department of Neonatology, Neonatal Biophysical Monitoring and Cardiopulmonary Therapies Research Unit, Poznan University of Medical Sciences, Poznan, Poland

**Keywords:** respiratory distress syndrome, preterm neonate, surfactant, less invasive surfactant administration (LISA), fraction of inspired oxygen, neonatal outcome

## Abstract

**Aim:** To establish the impact of oxygen requirement before surfactant (SF) and time from birth to SF administration on treatment outcomes in neonatal respiratory distress syndrome (RDS).

**Methods:** We conducted a *post-hoc* analysis of data from a prospective cohort study of 500 premature infants treated with less invasive surfactant administration (LISA). LISA failure was defined as the need for early (<72 h of life) mechanical ventilation (MV). Baseline clinical characteristic parameters, time to SF, and fraction of inspired oxygen (FiO_2_) prior to SF were all included in the multifactorial logistic regression model that explained LISA failure.

**Results:** LISA failed in 114 of 500 infants (22.8%). The median time to SF was 2.1 h (IQR: 0.8–6.7), and the median FiO_2_ prior to SF was 0.40 (IQR: 0.35–0.50). Factors significantly associated with LISA failure were FiO_2_ prior to SF (OR 1.03, 95% CI 1.01–1.04) and gestational age (OR 0.82, 95 CI 0.75–0.89); both *p* <0.001. Time to SF was not an independent risk factor for therapy failure (*p* = 0.528) or the need for MV at any time during hospitalization (*p* = 0.933).

**Conclusions:** The FiO_2_ before SF, but not time to SF, influences the need for MV in infants with RDS. While our findings support the relevance of FiO_2_ in SF prescription, better adherence to the recommended FiO_2_ threshold for SF (0.30) is required in daily practice.

## Introduction

Therapy with exogenous surfactant (SF) is a cornerstone in the management of neonatal respiratory distress syndrome (RDS). The treatment effect of SF is determined by the dose amount (1–4), the time of administration (5), and the severity of the neonate's condition. The latter is expressed by the fraction of inspired oxygen (FiO_2_), which is commonly used as a marker of the severity of respiratory distress (6).

The time of SF administration is widely viewed as critical, and the “the sooner – the better” concept is firmly established. This viewpoint is also supported by scientific evidence, including a meta-analysis of randomized trials, showing better outcomes with early vs. late SF administration (5). However, the strategy of prophylactic SF administration as soon as possible after birth, which had temporarily become the standard of treatment in the smallest babies (7), was discarded in later revisions of the European RDS Guidelines (8, 9). As shown in comparative trials, it was not more successful than early rescue administration of SF. The idea of an optimal “time window” for SF administration has been suggested (10), but the precise time boundaries have not been ultimately defined.

At present, the main criterion for SF administration is based on oxygen requirements, specifically the FiO_2_ level needed to maintain target saturation (11). In this context, a similar principle applies as in the case of time to SF: the sooner SF is given in the course of the disease (i.e., at lower oxygen requirements), the better. The FiO_2_ threshold of 0.30 has been considered optimal as a therapy trigger, and this strategy is endorsed by the most recent RDS Guidelines (11).

Given that a high SF dose (200 mg/kg) is a constant factor—as this dose is commonly used in Poland, the remaining two factors, time to SF and FiO_2_ prior to SF, can be considered potentially decisive for treatment outcomes. The primary goal of this analysis was to determine which of those two factors, as an independent factor, has a significant impact on the need for early mechanical ventilation (MV) as well as the need for MV at any time during the course of RDS. The secondary objective was to determine how soon after birth and at what FiO_2_ level SF is administered in daily practice.

## Materials and Methods

This was a *post hoc* analysis of data from a prospective cohort study of 500 premature infants with RDS. The study included babies who did not need primary intubation in the delivery room and were initially treated with non-invasive respiratory support combined with less invasive surfactant administration (LISA) at 31 tertiary-referral hospitals in Poland. The primary aim of the above study was to describe the implementation of a novel method of SF administration. Non-invasive respiratory support, such as continuous positive airway pressure (CPAP) with a minimum pressure of 6 cm H_2_O, bilevel CPAP, or nasal intermittent positive pressure ventilation (NIPPV), was used to maintain blood oxygen saturation in the target range of 90–94%. Standard criteria for surfactant were employed, as specified in the European Consensus Guidelines on the Management of Respiratory Distress Syndrome (9). Premedication was used at the discretion of the attending physician. The study protocol was approved by the Ethics Committee of Warsaw Medical University, and parents/legal guardians gave written consent for all diagnostic and therapeutic procedures in compliance with local law and practices. The data collection period was February 2018–March 2019. The current research is a follow-up of results from this prospective study, the primary findings of which were published elsewhere (12).

The key endpoint in this analysis was failure of the initial treatment plan encompassing non-invasive ventilation and LISA (LISA failure), necessitating early (before 72 h of life) MV. The coprimary efficacy endpoint was escalation of respiratory support to MV at any time during treatment.

To assess the impact of time to SF and FiO_2_ prior to SF on the occurrence of primary endpoints, multifactor models were prepared using the logistic regression method. In the initial models, the following explanatory variables were considered: time to SF, FiO_2_ prior to SF, sex, antenatal steroids, gestational age, birth weight, 5 min Apgar score and the maximum level of FiO_2_ in the delivery room. The final multivariate model was built using a stepwise backward elimination method. Akaike information criterion was employed to achieve the optimum balance of model goodness-of-fit and simplicity.

The baseline characteristics of the infants stratified by the primary outcome were compared using the Mann-Whitney test for continuous variables and χ2 or Fisher's exact tests, as appropriate, for dichotomous variables. All tests were two-tailed, and alpha = 0.05 was considered significant.

## Results

LISA procedures were performed in 500 babies, the majority of whom were non-premedicated (79%). Simultaneous breathing assistance included nasal CPAP (40%), bilevel CPAP (39%), and NIPPV used as primary or back-up procedure (24%). Non-invasive respiratory support combined with LISA failed in 114 of 500 infants (22.8%); these infants ultimately required MV in the first 72 h of life. The rate of MV at any time during hospitalization was 31%, adding 41 infants (8.2%) with late onset of MV to the 114 babies in which LISA failed (Table 1).

**Table 1:** Baseline characteristics of the study cohort stratified by primary outcome.

**Time to SF**	**A**.	**B**.		**C**.	
	**No MV**	**MV <72 h**	***P*-value**	**Any MV**	***P*-value**
	**(*n* = 345)**	**(*n* = 114)**	**A. vs. B**.	**(*n* = 155)**	**A. vs. C**.
Gestational age (weeks)	30.7 ± 2.4	28.9 ± 2.9	<0.001	28.6 ± 2.7	<0.001
Birth weight (g)	1455 (1111–1800)	1110 (855–1600)	<0.001	1040 (820–1425)	<0.001
Male sex	188 (55%)	68 (60%)	ns	86 (55%)	ns
Cesarean section	311 (90%)	103 (90%)	ns	141 (91%)	ns
Antenatal steroids	273 (79%)	87 (73%)	ns	118 (76%)	ns
Max FiO_2_ in the DR	0.3 (0.3–0.4)	0.38 (0.3–0.5)	0.005	0.4 (0.3–0.5)	0.002
Time from birth to SF	2.5 (1–8.7)	1.7 (0.7–4.5)	0.019	1.5 (0.6–3.5)	<0.001
FiO_2_ prior to SF	0.4 (0.35–0.5)	0.5 (0.4–0.6)	<0.001	0.45 (0.4–0.6)	0.006
SF dose (mg/kg)	192 (160–200)	187 (148–200)	ns	192 (151–200)	ns

*If not otherwise indicated, data are the mean ± SD or median (IQR). SF, surfactant; MV, mechanical ventilation; DR, delivery room*.

### FiO_2_ Prior to SF

**Figure 1:**
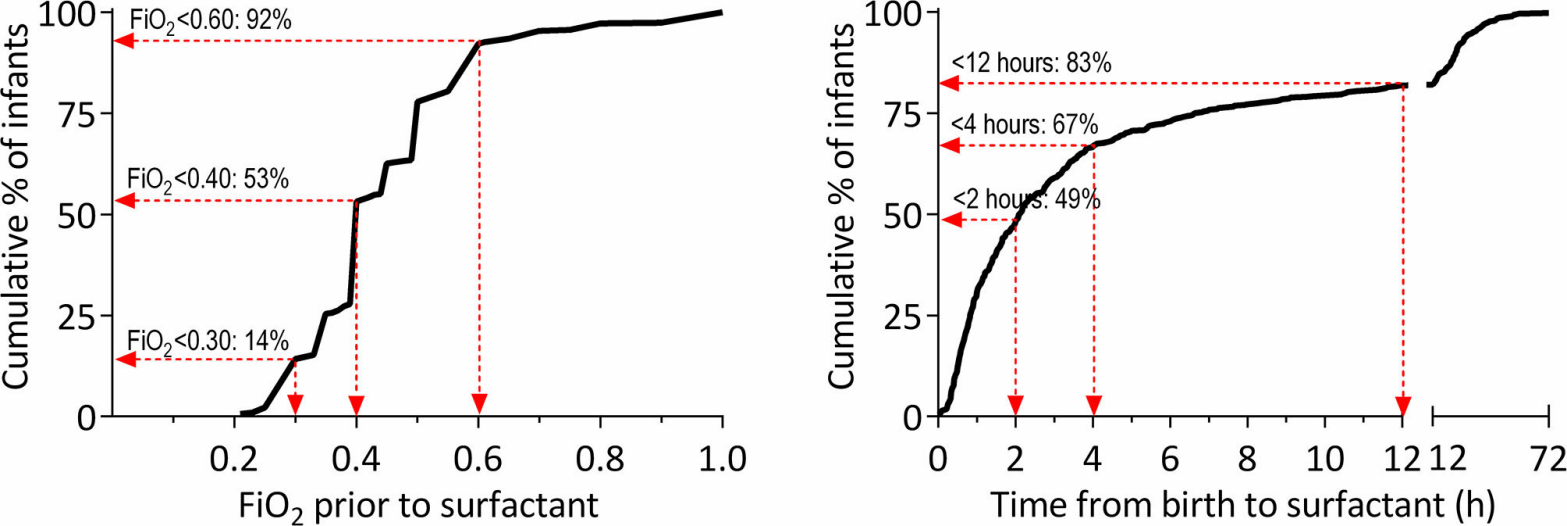
Histogram of cumulative distribution of FiO_2_ prior to SF (left) and time to SF (right).

The median FiO_2_ before SF was 0.40 (IQR: 0.35–0.50). As shown in Figure 1, approximately half of the infants (47%) were given SF at an FiO_2_ >0.40, and 8% were given SF at an FiO_2_ >0.60. No correlation was found between FiO_2_ prior to SF and time to SF (Pearson's *R* = 0.07; *p* = 0.126). Supplementary Figure 1 illustrates FiO_2_ before SF plotted against time to SF.

**Figure 2:**
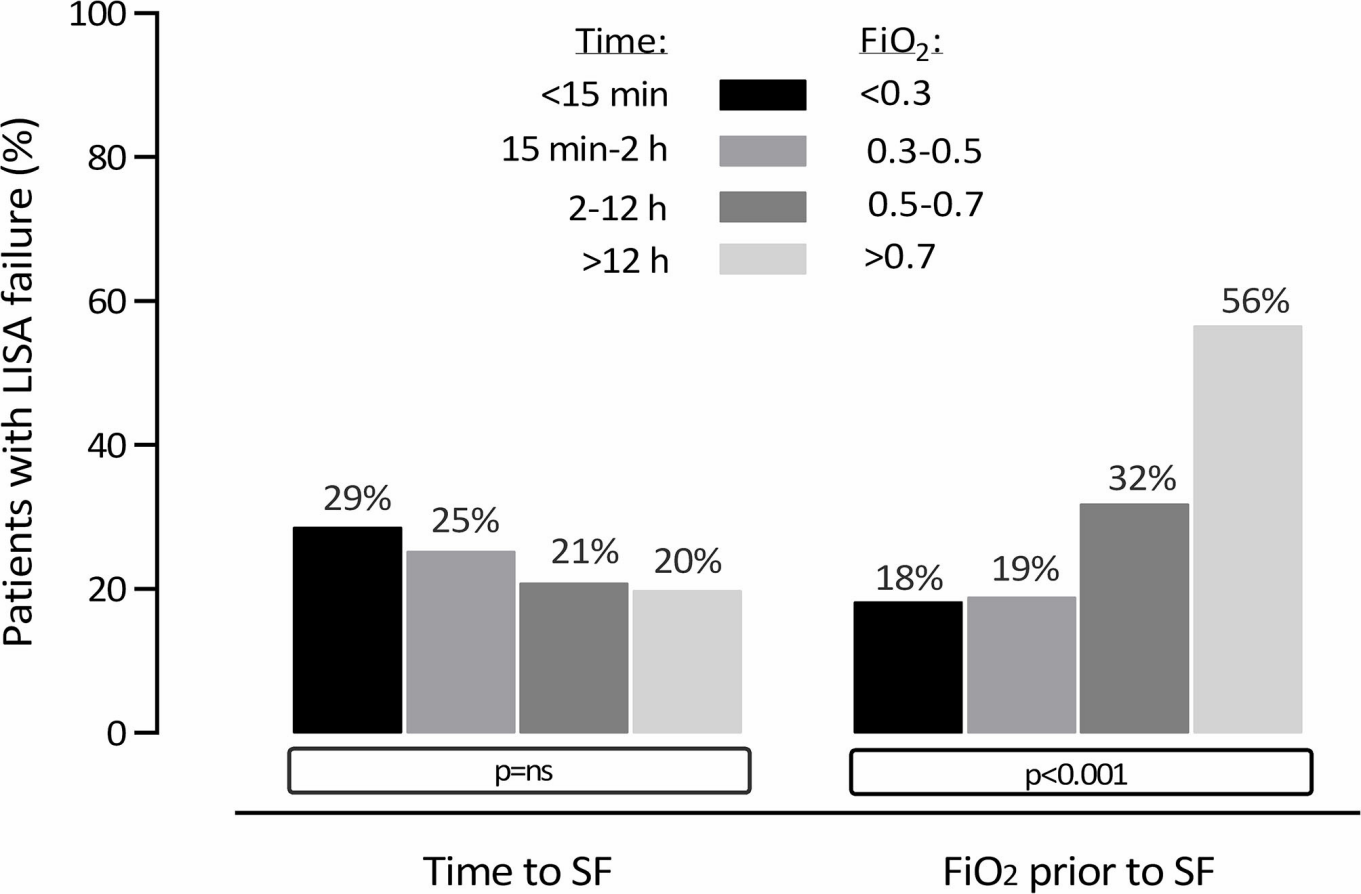
Need for MV <72 h of life indicating LISA failure, stratified by time to SF and FiO_2_ prior to SF.

The multivariate regression model confirmed a significant association between FiO_2_ prior to SF and LISA failure (Table 2). Administration of SF at higher levels of FiO_2_ was associated with a higher risk of MV before 72 h of life; the odds increased by 3% for every 0.01 increment of FiO_2_. A similar relationship was found for MV at any time during the disease; the odds of therapy escalation to MV increased by 2.4% with every 0.01 increase in FiO_2_ prior to SF (Table 3). LISA failure rates varied according to FiO_2_ before SF, ranging from 18% (at an FiO_2_ <0.30) to 56% (at an FiO_2_ >0.70) (Figure 2).

**Table 2:** Logistic regression analysis examining the prediction failure of LISA combined with CPAP.

	**MV before 72 h of life**					
	**Initial multivariate model**			**Final multivariate model**		
	**OR**	**95% CI**	***P***	**OR**	**95%CI**	***P***
Gestation (weeks)	0.78	0.66–0.92	0.003	0.82	0.74–0.88	<0.001
Birth weight (100 g)	1.02	0.94–1.10	ns	—	—	—
5 min Apgar	0.85	0.69–1.03	0.098	0.84	0.70–1.02	0.090
Sex (male)	1.19	0.76–1.87	ns	—	—	—
Antenatal steroids	0.64	0.38–1.10	0.101	0.63	0.38–1.08	0.091
Max FiO_2_ in the delivery room	1.00	0.99–1.01	ns	—	—	—
FiO_2_ prior to SF	1.03	1.01–1.04	<0.001	1.03	1.01–1.04	<0.001
Time from birth to SF (h)	1.01	0.98–1.03	ns	—	—	—

*SF, surfactant; MV, mechanical ventilation*.

**Table 3:** Logistic regression analysis examining variables influencing the need for MV at any time during hospitalization.

	**Any MV**					
	**Initial multivariate model**			**Final multivariate model**		
	**OR**	**95% CI**	***P***	**OR**	**95%CI**	***P***
Gestation (weeks)	0.75	0.64–0.87	<0.001	0.72	0.66–0.79	<0.001
Birth weight (100 g)	0.98	0.90–1.06	ns	—	—	—
5 min Apgar	0.93	0.77–1.12	ns	—	—	—
Sex (male)	0.95	0.62–1.44	ns	—	—	—
Antenatal steroids	0.68	0.41–1.15	0.143	0.68	0.41–1.14	0.138
Max FiO_2_ in the delivery room	1.00	0.99–1.01	ns	—	—	—
FiO_2_ prior to SF	1.02	1.01–1.04	0.003	1.02	1.01–1.04	<0.001
Time from birth to SF (h)	1.00	0.98–1.02	ns	—	—	—

*SF, surfactant; MV, mechanical ventilation*.

FiO_2_ before surfactant had a weak although statistically significant correlation with maximum FiO_2_ in the delivery room (Spearman's rho = 0.28; *p* < 0.001).

### Time to SF Administration

The median time from birth to SF administration was 2.1 h (IQR: 0.8–6.7). Time to SF was not an independent risk factor for LISA failure (*p* = 0.528) or the need for MV at any time during the disease (*p* = 0.933). Additionally, as shown in Figure 2, the rates of LISA failure stratified by time to SF did not show statistically significant variability.

### Clinical Outcomes

Infants with LISA failure required repeated doses of surfactant more often than those with LISA success (48 vs. 6%, *p* < 0.001).

The need for MV <72 h of life was also associated with higher rates of intraventricular hemorrhages (IVH), including severe IVH (11 vs. 2%, *p* < 0.001) and increased in-hospital mortality (17 vs. 1%, *p* < 0.001) (Supplementary Table 1).

Compared to lower (≤0.30) FiO_2_ levels before SF, higher FiO_2_ (>0.60) was associated with more frequent LISA failure (46 vs. 20%; *p* = 0.007) and longer duration of mechanical ventilation (mean 7 vs. 3 days; *p* = 0.006) (Supplementary Table 2).

## Discussion

We have shown that FiO_2_ prior to SF administration, but not time from birth to SF, influences the need for MV in infants with RDS. This appears to be an important finding in the era of minimally invasive SF therapy, with potential practical implications.

The timing of SF administration during RDS management is a well-known treatment success factor. Early (within the first 2 h of life) vs. delayed SF treatment is associated with lower neonatal mortality and a lower risk of acute lung injury, air leak syndromes, and BPD (10, 13). In our analysis, however, timing had no impact on our primary outcome measure, i.e., the need for MV. Although earlier recommendations strongly emphasized the role of timing of SF and even advocated its administration within the first 15 min of life in “almost all babies <26 weeks of gestation” (7), they were based on findings from studies conducted when early CPAP was not a standard practice and antenatal steroids were used less frequently. Furthermore, in studies investigating the impact of SF timing on survival and complication rates, babies were given medication via an endotracheal tube, either with short-term manual ventilation after dosing or while remaining on MV (10, 13). As minimally invasive SF administration has become a new standard of care, it appears critical to understand which factors influence the therapy's success with this novel approach.

In the recent ESTHER study by Raschetti and colleagues (14), the use of ultrasound imaging to reduce the time to SF had little effect on the rate of MV, although it did affect its duration. In terms of timing, current guidelines recommend that SF be given “early in the course of the disease” (11). In daily practice, SF is usually given within hours from birth rather than minutes. In our cohort, the median time to SF was 2.1 h, and ~60% of the infants received SF within the first 3 h of life. This percentage was somewhat lower than the corresponding ESTHER trial's 71%. Nonetheless, the lack of an impact of timing on the primary outcome shows that SF has been applied within the optimal time frame.

It seems obvious that the severity of respiratory distress affects the efficacy of SF therapy. In the logistic regression model, however, it turned out that the oxygen demand prior to SF administration rather than the initial FiO_2_ significantly correlated with LISA failure. This finding suggests that not the early postbirth severity of respiratory distress but rather the lack of improvement/stabilization in the first hours of life is a significant risk factor for MV.

In everyday practice, FiO_2_ is a widely used parameter for assessing the severity of respiratory failure and tracking the dynamics of RDS. Since non-invasive ventilation is presently used to stabilize more than 80% of preterm newborns (15, 16), predictors of CPAP failure take on added relevance. Dargaville et al. (17) and Gulczyńska et al. (18) found that increased oxygen demand exceeding 30% in the first hours of life is associated with likely therapy failure. In our study, we confirmed the association of FiO_2_ with the need for therapy escalation and demonstrated that as the oxygen requirement prior to SF increases, so does the risk of MV. Therefore, our findings emphasize the significance of the FiO2 level in deciding whether to prescribe SF. In this context, the cohort's median FiO_2_ prior to SF of 0.40 must be considered suboptimal when compared to the recommended criterion of 0.30 (11).

While our data confirm the relevance of FiO_2_ in SF prescription, it is crucial to note that increased oxygen demand is only one of several factors that determine the severity of RDS, and patients may benefit from other monitoring methods. These may include techniques such as serial lung ultrasound examinations, transcutaneous CO_2_ (TcpCO_2_), electric diaphragm activity monitoring, electric impedance tomography, cerebral tissue oxygenation measurement and SF biological tests (6, 14, 19–23). Prospects for the future include determining the metabolomic profile of blood, urine, or exhaled air (23). All of these factors can hasten the SF decision or encourage early measures unrelated to SF that reduce the risk of MV, such as increasing end-expiratory pressure, switching from nCPAP to NIPPV, or considering special therapies that improve the efficacy of respiratory support, such as heliox (23–26).

Our findings should be viewed with caution because the need for MV, which was our study's endpoint, may have multifactorial causality. It may be associated not only with the deterioration of lung function but also with insufficient respiratory drive or hemodynamic disturbances. Additionally, SF dosage is known to significantly influence the treatment effect, as has been demonstrated in previous trials (1, 4, 27). However, in our population, this parameter showed little variation, as all patients received an SF dose close to 200 mg/kg. As a result, this parameter was not the focus of this analysis.

Among other limitations, our dataset lacks information on changes in post-surfactant FiO_2_ or respiratory support parameters such as mean airway pressure. Furthermore, because our study only included newborns treated with LISA, we were unable to compare neonatal outcomes in infants requiring high FiO_2_, who received SF using LISA to those who received it through endotracheal tube.

The results of the study indicate that our SF administration practice is close to optimal in terms of dosage and timing but that stricter adherence to the recommended FiO_2_ threshold for SF is needed. The treatment response rate might possibly be improved by extending SF treatment to those patients who, despite not requiring more than 30% oxygen, show noticeable lesions on lung ultrasound imaging. However, the latter thesis must be verified in further studies.

## Data Availability Statement

The raw data supporting the conclusions of this article will be made available by the authors, without undue reservation.

## Ethics Statement

The studies involving human participants were reviewed and approved by Ethics Committee of Warsaw Medical University. Written informed consent to participate in this study was provided by the participants' legal guardian/next of kin.

## Author Contributions

All authors listed have made a substantial, direct and intellectual contribution to the work, and approved it for publication.

## Funding

The authors declare that this study received funding from Chiesi Poland, a subsidiary of Chiesi Farmaceutici, Italy. Dr. RH (MD and employee of Chiesi) was involved in co-designing the study and preparation of the manuscript. Chiesi funding covered the costs of data collection and statistical analysis, which were handled by an independent contract research organization, as well as an open-access publishing fee.

## Conflict of Interest

PKru and PKra received speaker honoraria from Chiesi Poland and travel grants for participation in scientific conferences. TS received honoraria from Chiesi Poland for lecturing and participation on advisory boards. RH is employed by Chiesi Poland, the sponsor of the analyzed study.

## Publisher's Note

All claims expressed in this article are solely those of the authors and do not necessarily represent those of their affiliated organizations, or those of the publisher, the editors and the reviewers. Any product that may be evaluated in this article, or claim that may be made by its manufacturer, is not guaranteed or endorsed by the publisher.

## Supplementary Material

Supplementary Figure 1 Time to surfactant plotted against FiO_2_ before surfactant.
