# Supplementary material for: FiO2 Before Surfactant, but Not Time to Surfactant, Affects Outcomes in Infants With Respiratory Distress Syndrome
Source: Front Pediatr. 2021 Oct 4;9:734696. doi: 10.3389/fped.2021.734696 (PMC8520978; doi:10.3389/fped.2021.734696)
Supplement: Supplementary file 1 [file Table_1.pdf]

Supplementary Table 1S. Clinical outcomes in infants requiring MV <72 h of life (LISA failure) versus those who did not require MV (LISA success)

	**LISA failure** (n=114)	**LISA success** (n=385)	***P***
Duration of non-invasive ventilation; days	6 (2 - 23)	7 (4 - 21)	0.075
BPD	44 (39%)	114 (30%)	0.089
BPD moderate/severe	18 (16%)	55 (14%)	ns
IVH	40 (35%)	54 (14%)	<0.001
IVH grade 3 or 4	13 (11%)	7 (2%)	<0.001
In-hospital mortality	20 (17%)	3 (1%)	<0.001

BPD = bronchopulmonary dysplasia, IVH = intraventricular hemorrhage.
