# Supplementary material for: FiO2 Before Surfactant, but Not Time to Surfactant, Affects Outcomes in Infants With Respiratory Distress Syndrome
Source: Front Pediatr. 2021 Oct 4;9:734696. doi: 10.3389/fped.2021.734696 (PMC8520978; doi:10.3389/fped.2021.734696)
Supplement: Supplementary file 2 [file Table_2.pdf]

Supplementary Table 2S. Baseline characteristics and clinical outcomes in infants stratified by FiO_2 before surfactant.

	**„Low" FiO_2 (≤0.30)** (n=71)	**„High" FiO_2 (>0.60)** (n=39)	***P***
**Baseline characteristics**			
Gestational age (weeks)	29.6 ±2.6	29.6 ±3.4	ns
Birth weight (g)	1288 ±435	1384 ±743	ns
Male sex	40 (56%)	21 (54%)	ns
5 min Apgar			
Cesarean section	65 (91%)	35 (90%)	ns
Antenatal steroids	59 (83%)	28 (72%)	ns
Maximum FiO_2 in the Delivery room	0.3 (0.3 – 0.3)	0.4 (0.3 – 0.8)	<0.001
Time from birth to SF	2.17 (0.75 – 5.00)	1.52 (0.64 – 3.17)	ns
**Clinical outcomes**			
MV < 72 h of life	14 (20%)	18 (46%)	0.007
Duration of MV (days)	3.0 ±8.2	6.8 ±12.5	0.006
Duration of non-invasive ventilation (days)	7 (4 – 21)	7 (3 – 26)	ns
BPD	18 (25%)	16 (41%)	ns
BPD moderate or severe	9 (13%)	7 (18%)	ns
IVH	22 (31%)	6 (15%)	ns
IVH grade 3 or 4	6 (8%)	0 (0%)	0.087
In-hospital mortality	4 (6%)	5 (13%)	ns

If not otherwise indicated, data are the mean ± SD or median (IQR).
BPD = bronchopulmonary dysplasia, IVH = intraventricular hemorrhage.
