# Supplementary figures and images for: FiO2 Before Surfactant, but Not Time to Surfactant, Affects Outcomes in Infants With Respiratory Distress Syndrome
Source: Front Pediatr. 2021 Oct 4;9:734696. doi: 10.3389/fped.2021.734696 (PMC8520978; doi:10.3389/fped.2021.734696)

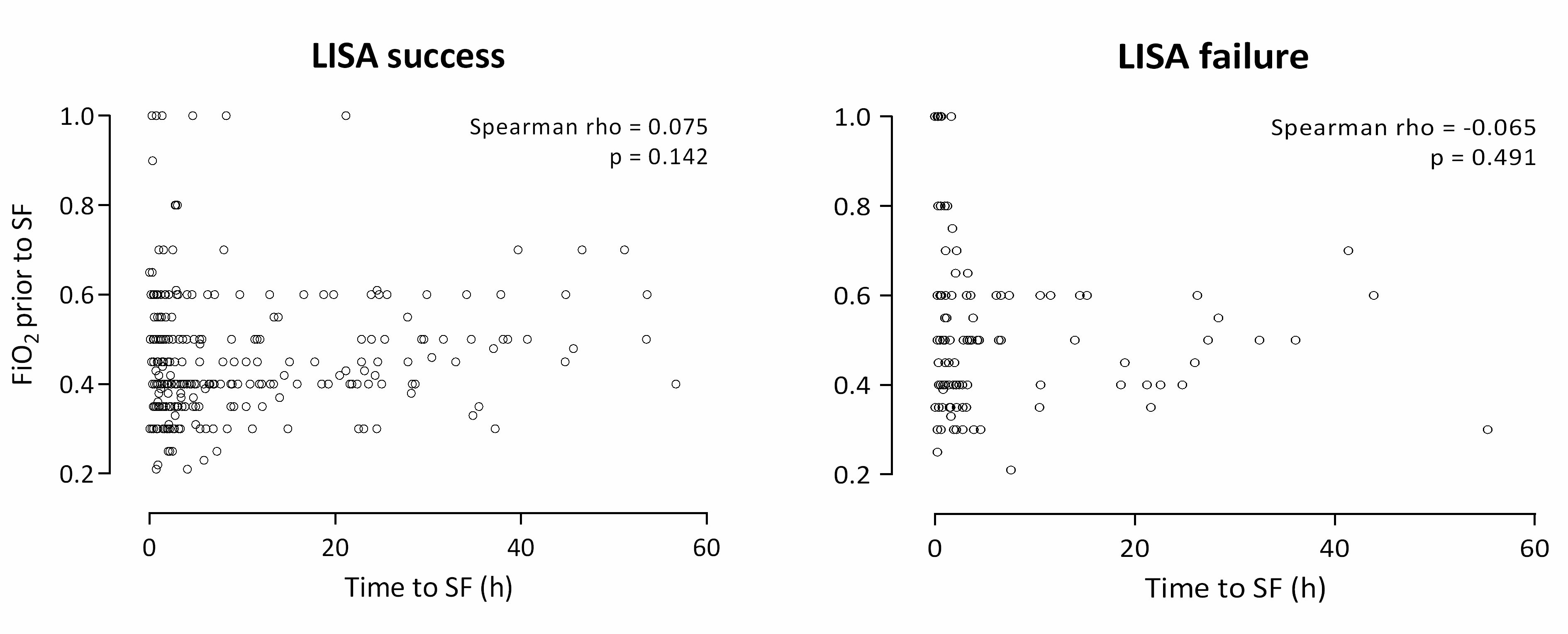

Supplement: Supplementary Figure 1 — Time to surfactant plotted against FiO2 before surfactant. [file Image_1.TIF]
